# Supplementary material for: Spotlight on islands: on the origin and diversification of an ancient lineage of the Italian wall lizard Podarcis siculus in the western Pontine Islands
Source: Sci Rep. 2018 Oct 11;8:15111. doi: 10.1038/s41598-018-33326-w (PMC6181948; doi:10.1038/s41598-018-33326-w)
Supplement: Supplementary file 1 — Supplementary Information [file 41598_2018_33326_MOESM1_ESM.docx]

**Spotlight on islands: on the origin and diversification of an ancient lineage of the Italian wall lizard *Podarcis siculus* in the western Pontine Islands**

Senczuk Gabriele, Havenstein Katja, Milana Valentina, Ripa Chiara, De Simone Emanuela, Tiedemann Ralph, and Castiglia Riccardo

**Supplementary Information**

**Table S1.** Locality names with their relative geographic coordinates, sample size, and frequencies of each mitochondrial and nuclear haplotype.

|  | | |  | | | |  | |  | |  | | **mtDNA** | | | ***mc1r*** | | | | | | | *** β-fibint7*** | | | |  |
| --- | --- | --- | --- | --- | --- | --- | --- | --- | --- | --- | --- | --- | --- | --- | --- | --- | --- | --- | --- | --- | --- | --- | --- | --- | --- | --- | --- |
| **Code** | | | **Locality** | | | | **Lat.** | | **Long.** | | **Voucher** | | **n Hap. n Hap. n Hap.** | | | | | | | | | | | | | | |
| 6S | Nebrodi | | | | 37.8736 | | | 14.6256 | | RS15 | | 2 | | - | | | / | | / | | | 2 | | (F20; F25) | |  |  |
|  |  | | | |  | | |  | | RS16 | |  | | - | | | / | | / | | | 2 | | F20 | |  |  |
| 14S | Zingaro | | | | 38.0935 | | | 12.7981 | | RS48 | | 2 | | - | | | 2 | | M9 | | | 2 | | F20 | |  |  |
|  |  | | | |  | | |  | | RS49 | |  | | - | | | 2 | | M9 | | | 2 | | F20 | |  |  |
| 15S | Monte Cofano | | | | 38,105 | | | 12,677 | | RS54 | | 1 | | - | | | / | | / | | | / | | / | |  |  |
| 16S | Favignana | | | | 37.9154 | | | 12.3201 | | RS57 | | 1 | | - | | | / | | M9 | | | 2 | | F21 | |  |  |
| 19S | Rilievo Fiume | | | | 37.8836 | | | 12.5280 | | RS103 | | 3 | | - | | | / | | / | | | 2 | | F24 | |  |  |
|  |  | | | |  | | |  | | RS104 | |  | | - | | | 2 | | M9 | | | / | | / | |  |  |
|  |  | | | |  | | |  | | RS105 | |  | | - | | | 2 | | M9 | | | / | | / | |  |  |
| 25S | Siculiana | | | | 37.3407 | | | 13.3914 | | RS157 | | 2 | | - | | | 2 | | M9 | | | / | | / | |  |  |
|  |  | | | |  | | |  | | RS157b | |  | | - | | | 2 | | M9 | | | / | | / | |  |  |
| 31S | Sughereta | | | | 37,071 | | | 14,439 | | RS223 | | 1 | | - | | | / | | / | | | / | | / | |  |  |
| 36S | Vendicari | | | | 36.8034 | | | 15.0958 | | RS32 | | 2 | | - | | | 2 | | M9 | | | 2 | | F20 | |  |  |
|  |  | | | |  | | |  | | RS36 | |  | | - | | | 2 | | (M9; M14) | | | 2 | | (F20; F23) | |  |  |
| 39S | Cassibile | | | | 36.9820 | | | 15.1649 | | RS165 | | 1 | | - | | | 2 | | (M9; M14) | | | 2 | | (F21; F22) | |  |  |
| 44S | Lentino | | | | 37.3339 | | | 14.9994 | | RS169 | | 2 | | - | | | 2 | | (M9; M14) | | | 2 | | F24 | |  |  |
|  |  | | | |  | | |  | | RS170 | |  | | - | | | 2 | | M9 | | | / | | / | |  |  |
| 45S | Foce Simeto | | | | 37,378 | | | 15,035 | | RS196 | | 1 | | - | | | / | | / | | | / | | / | |  |  |
| 48S | Trecastagne | | | | 37,621 | | | 15,091 | | RS190 | | 1 | | - | | | / | | / | | | / | | / | |  |  |
| 52S | Taormina | | | | 37.8510 | | | 15.2980 | | RS06 | | 2 | | - | | | 2 | | M9 | | | 2 | | (F20; F24) | |  |  |
|  |  | | | |  | | |  | | RS07 | |  | | - | | | 2 | | (M9; M5) | | | / | | / | |  |  |
| 53S | Isola bella | | | | 37.8499 | | | 15.3003 | | RS05 | | 1 | | - | | | 2 | | (M9; M26) | | | 2 | | F20 | |  |  |
| 55S | Gole Alcantara | | | | 37.8715 | | | 15.1659 | | RS09 | | 2 | | - | | | 2 | | M9 | | | / | | / | |  |  |
|  |  | | | |  | | |  | | RS11 | |  | | - | | | / | | / | | | 2 | | (F20; F24) | |  |  |
| 1CL | Gerace | | | | 38,276 | | | 16,220 | | RCL140 | | 1 | | - | | | / | | / | | | 2 | | (F26; F27) | |  |  |
| 2CL | Mammola | | | | 38,364 | | | 16,243 | | RCL141 | | 2 | | - | | | / | | / | | | 2 | | (F20; F29) | |  |  |
|  |  | | | |  | | |  | | RCL142 | |  | | - | | | / | | / | | | 2 | | F18 | |  |  |
| 4CL | Rende | | | | 39.3306 | | | 16.18489 | | RCL113 | | 2 | | - | | | 2 | | M10 | | | 2 | | (F13; F28) | |  |  |
|  |  | | | |  | | |  | | RCL114 | |  | | - | | | 2 | | (M10; M13) | | | 2 | | F16 | |  |  |
| 5CL | Mileto | | | | 38,608 | | | 16,061 | | RCL136 | | 2 | | - | | | / | | / | | | 2 | | (F30; F31) | |  |  |
|  |  | | | |  | | |  | | RCL137 | |  | | - | | | / | | / | | | 2 | | (F28; F15) | |  |  |
| 8CL | Steccato di Cutro | | | | 38,938 | | | 16,910 | | RCL148 | | 2 | | - | | | / | | / | | | 2 | | F28 | |  |  |
|  |  | | | |  | | |  | | RCL150 | |  | | - | | | / | | / | | | 2 | | F28 | |  |  |
| 11CL | Falerna | | | | 39.0004 | | | 16.1779 | | RCL119 | | 1 | | - | | | / | | / | | | / | | / | |  |  |
| 13CL | Belmonte | | | | 39.1609 | | | 16.0829 | | RCL115 | | 2 | | - | | | / | | / | | | / | | / | |  |  |
|  |  | | | |  | | |  | | RCL116 | |  | | - | | | / | | / | | | / | | / | |  |  |
| 14CL | Fiumefreddo | | | | 39.2317 | | | 16.0749 | | RCL111 | | 2 | | - | | | 2 | | (M4; M9) | | | 2 | | F14 | |  |  |
|  |  | | | |  | | |  | | RCL112 | |  | | - | | | 2 | | (M10; M11) | | | 2 | | F28 | |  |  |
| 1P | Gravina | | | | 40.7960 | | | 16.4231 | | RP06 | | 1 | | - | | | / | | / | | | 2 | | F17 | |  |  |
| 2P | Rosa Marina | | | | 40.7925 | | | 17.5562 | | RP07 | | 1 | | - | | | 2 | | M21 | | | / | | / | |  |  |
| 3P | S. Domino | | | | 42.1170 | | | 15.4944 | | RP08 | | 1 | | - | | | 2 | | M21 | | | 2 | | F19 | |  |  |
| 1C | Rovine di Velia | | | | 40.1616 | | | 15.1636 | | RC14 | | 2 | | - | | | 2 | | M24 | | | 2 | | F12 | |  |  |
|  |  | | | |  | | |  | | RC15 | |  | | - | | | 2 | | M24 | | | 2 | | F11 | |  |  |
| 4C | Punta Licosa | | | | 40.2342 | | | 14.9454 | | RC10 | | 2 | | - | | | / | | / | | | / | | / | |  |  |
|  |  | | | |  | | |  | | RC11 | |  | | - | | | 2 | | M24 | | | 2 | | F10 | |  |  |
| 5C | Capaccio | | | | 40.4172 | | | 15.0945 | | RC05 | | 1 | | - | | | 2 | | (M21; M24) | | | / | | / | |  |  |
| 6C | Roscigno | | | | 40.4143 | | | 15.3349 | | RC02 | | 1 | | - | | | 2 | | (M21; M24) | | | / | | / | |  |  |
|  |  | | | |  | | |  | | RC03 | |  | | - | | | / | | / | | | 2 | | (F8;F10) | |  |  |
| 7C | R.N. Sale Tanagro | | | | 40.5198 | | | 14.9238 | | RC19 | | 2 | | - | | | / | | / | | | 2 | | F10 | |  |  |
|  |  | | | |  | | |  | | RC20 | |  | | - | | | / | | / | | | 2 | | (F10; F42) | |  |  |
| 1L | Suio Terme | | | | 41.2961 | | | 13.8677 | | RL120 | | 1 | | - | | | 2 | | (M9; M24) | | | / | | / | |  |  |
| 2L | Sperlonga | | | | 41.2606 | | | 13.4477 | | RL46 | | 3 | | - | | | 2 | | (M9; M25) | | | / | | / | |  |  |
|  |  | | | |  | | |  | | RL47 | |  | | - | | | 2 | | (M9; M24) | | | 2 | | F33 | |  |  |
|  |  | | | |  | | |  | | RL48 | |  | | - | | | 2 | | (M9; M6) | | | / | | / | |  |  |
| 3L | Circeo foresta | | | | 41.3545 | | | 13.0575 | | RL19 | | 1 | | - | | | 2 | | M9 | | | / | | / | |  |  |
| 4L | Lago dei Monaci | | | | 41.4050 | | | 12.876 | | RL20 | | 5 | | - | | | 2 | | (M8; M9) | | | / | | / | |  |  |
|  |  | | | |  | | |  | | RL50 | |  | | - | | | 2 | | M9 | | | 2 | | F6 | |  |  |
|  |  | | | |  | | |  | | RL51 | |  | | - | | | 2 | | M9 | | | 2 | | (F35; F36) | |  |  |
|  |  | | | |  | | |  | | RL52 | |  | | - | | | 2 | | (M8; M9) | | | / | | / | |  |  |
|  |  | | | |  | | |  | | RL53 | |  | | - | | | 2 | | (M8; M9) | | | / | | / | |  |  |
| 5L | Zannone | | | | 40.9682 | | | 13.0531 | | RL161 | | 8 | | H2 | | | 2 | | (M30; M31) | | | / | | / | |  |  |
|  |  | | | |  | | |  | | RL163 | |  | | H1 | | | 2 | | (M9; M31) | | | 2 | | F3 | |  |  |
|  |  | | | |  | | |  | | RL164 | |  | | H3 | | | 2 | | (M9; M31) | | | 2 | | F2 | |  |  |
|  |  | | | |  | | |  | | RL166 | |  | | H5 | | | 2 | | M9 | | | 2 | | F1 | |  |  |
|  |  | | | |  | | |  | | RL168 | |  | | H2 | | | 2 | | (M31; M33) | | | 2 | | F2 | |  |  |
|  |  | | | |  | | |  | | RL169 | |  | | H4 | | | 2 | | (M9; M31) | | | 2 | | F2 | |  |  |
|  |  | | | |  | | |  | | RL170 | |  | | H6 | | | / | | / | | | / | | / | |  |  |
|  |  | | | |  | | |  | | RL171 | |  | | H7 | | | 2 | | (M3; M31) | | | 2 | | F2 | |  |  |
| 6L | Gavi | | | | 40.9342 | | | 12.9993 | | RL95 | | 20 | | H24 | | | 2 | | M31 | | | 2 | | (F2; F1) | |  |  |
|  |  | | | |  | | |  | | RL96 | |  | | H24 | | | 2 | | (M23; M31) | | | / | | / | |  |  |
|  |  | | | |  | | |  | | RL97 | |  | | H27 | | | 2 | | (M23; M29) | | | 2 | | (F2; F1) | |  |  |
|  |  | | | |  | | |  | | RL98 | |  | | H24 | | | 2 | | (M23; M31) | | | 2 | | F2 | |  |  |
|  |  | | | |  | | |  | | RL99 | |  | | H25 | | | 2 | | (M23; M31) | | | 2 | | F1 | |  |  |
|  |  | | | |  | | |  | | RL100 | |  | | H24 | | | 2 | | M23 | | | / | | / | |  |  |
|  |  | | | |  | | |  | | RL101 | |  | | H26 | | | 2 | | (M30; M31) | | | / | | / | |  |  |
|  |  | | | |  | | |  | | RL102 | |  | | H28 | | | 2 | | (M9; M31) | | | / | | / | |  |  |
|  |  | | | |  | | |  | | RL103 | |  | | H24 | | | 2 | | (M9; M23) | | | / | | / | |  |  |
|  |  | | | |  | | |  | | RL104 | |  | | H27 | | | 2 | | (M23; M31) | | | / | | / | |  |  |
|  |  | | | |  | | |  | | RL105 | |  | | H24 | | | 2 | | M23 | | | / | | / | |  |  |
|  |  | | | |  | | |  | | RL106 | |  | | H24 | | | 2 | | (M15; M19) | | | 2 | | F2 | |  |  |
|  |  | | | |  | | |  | | RL107 | |  | | H24 | | | 2 | | M23 | | | / | | / | |  |  |
|  |  | | | |  | | |  | | RL109 | |  | | H24 | | | 2 | | (M17; M23) | | | 2 | | F1 | |  |  |
|  |  | | | |  | | |  | | RL110 | |  | | H24 | | | 2 | | (M29; M30) | | | 2 | | F2 | |  |  |
|  |  | | | |  | | |  | | RL111 | |  | | H24 | | | 2 | | M31 | | | / | | / | |  |  |
|  |  | | | |  | | |  | | RL113b | |  | | H24 | | | 2 | | (M9; M31) | | | / | | / | |  |  |
|  |  | | | |  | | |  | | RL114 | |  | | H24 | | | 2 | | (M9; M16) | | | / | | / | |  |  |
|  |  | | | |  | | |  | | RL117 | |  | | H24 | | | 2 | | M31 | | | / | | / | |  |  |
|  |  | | | |  | | |  | | RL119 | |  | | H24 | | | 2 | | M23 | | | / | | / | |  |  |
| 7aL | Ponza (Forna) | | | | 40.9192 | | | 12.9647 | | RL08 | | 18 | | H24 | | | 2 | | (M31; M33) | | | 2 | | F2 | |  |  |
|  |  | | | |  | | |  | | RL09 | |  | | H16 | | | 2 | | M33 | | | 2 | | F2 | |  |  |
|  |  | | | |  | | |  | | RL10 | |  | | H16 | | | 2 | | (M31; M33) | | | / | | / | |  |  |
|  |  | | | |  | | |  | | RL28 | |  | | H29 | | | 2 | | M31 | | | 2 | | F1 | |  |  |
|  |  | | | |  | | |  | | RL29 | |  | | H22 | | | 2 | | (M31; M32) | | | / | | / | |  |  |
| 7bL | Ponza (M. Guardia) | | | | 40.8887 | | | 12.9591 | | RL55 | |  | | H16 | | | 2 | | (M9; M33) | | | / | | / | |  |  |
|  |  | | | |  | | |  | | RL57 | |  | | H18 | | | 2 | | (M33; M34) | | | 2 | | (F1; F3) | |  |  |
|  |  | | | |  | | |  | | RL58 | |  | | H23 | | | 2 | | (M9; M33) | | | 2 | | F41 | |  |  |
|  |  | | | |  | | |  | | RL59 | |  | | H16 | | | 2 | | M33 | | | 2 | | F2 | |  |  |
|  |  | | | |  | | |  | | RL60 | |  | | H30 | | | 2 | | (M9; M33) | | | 2 | | F2 | |  |  |
|  |  | | | |  | | |  | | RL61 | |  | | H31 | | | 2 | | M33 | | | 2 | | F2 | |  |  |
|  |  | | | |  | | |  | | RL62 | |  | | H16 | | | 2 | | M31 | | | / | | / | |  |  |
|  |  | | | |  | | |  | | RL63 | |  | | H17 | | | 2 | | (M9; M31) | | | 2 | | F41 | |  |  |
|  |  | | | |  | | |  | | RL64 | |  | | H24 | | | 2 | | (M9; M33) | | | / | | / | |  |  |
|  |  | | | |  | | |  | | RL65 | |  | | H16 | | | 2 | | (M31; M33) | | | 2 | | F3 | |  |  |
|  |  | | | |  | | |  | | RL67 | |  | | H21 | | | 2 | | (M23;M31) | | | 2 | | F3 | |  |  |
|  |  | | | |  | | |  | | RL68 | |  | | H24 | | | 2 | | M31 | | | / | | / | |  |  |
|  |  | | | |  | | |  | | RL69 | |  | | H19 | | | 2 | | M31 | | | / | | / | |  |  |
| 7cL | Ponza (FM) | | | | 40.8941 | | | 12.9729 | | RL130 | | 5 | | H16 | | | 2 | | (M31; M33) | | | / | | / | |  |  |
|  |  | | | |  | | |  | | RL132 | |  | | H20 | | | 2 | | (M31; M33) | | | 2 | | (F4; F41) | |  |  |
|  |  | | | |  | | |  | | RL133 | |  | | H16 | | | 2 | | (M31; M21) | | | 2 | | F41 | |  |  |
|  |  | | | |  | | |  | | RL134 | |  | | H10 | | | 2 | | (M9; M33) | | | 2 | | F3 | |  |  |
|  |  | | | |  | | |  | | RL135 | |  | | H21 | | | / | | / | | | / | | / | |  |  |
| 8L | Palmarola | | | | 40.9350 | | | 12.8541 | | RL136 | | 10 | | H16 | | | 2 | | M30 | | | / | | / | |  |  |
|  |  | | | |  | | |  | | RL138 | |  | | H12 | | | 2 | | M30 | | | / | | / | |  |  |
|  |  | | | |  | | |  | | RL139 | |  | | H14 | | | 2 | | M9 | | | / | | / | |  |  |
|  |  | | | |  | | |  | | RL140 | |  | | / | | | 2 | | (M30; M33) | | | 2 | | F3 | |  |  |
|  |  | | | |  | | |  | | RL141 | |  | | H13 | | | 2 | | M31 | | | 2 | | F2 | |  |  |
|  |  | | | |  | | |  | | RL142 | |  | | H9 | | | 2 | | (M30; M31) | | | 2 | | F2 | |  |  |
|  |  | | | |  | | |  | | RL143 | |  | | H8 | | | 2 | | (M9; M31) | | | 2 | | F2 | |  |  |
|  |  | | | |  | | |  | | RL144 | |  | | H15 | | | 2 | | M9 | | | 2 | | (F2; F1) | |  |  |
|  |  | | | |  | | |  | | RL145 | |  | | H11 | | | 2 | | (M17; M18) | | | 2 | | (F2; F3) | |  |  |
|  |  | | | |  | | |  | | RL146 | |  | | H8 | | | 2 | | M9 | | | / | | / | |  |  |
|  |  | | | |  | | |  | | RL148 | |  | | / | | | 2 | | (M31; M32) | | | 2 | | (F5; F3) | |  |  |
|  |  | | | |  | | |  | | RL149 | |  | | H9 | | | 2 | | (M9; M31) | | | / | | / | |  |  |
| 9L | Foglino | | | | 41.4726 | | | 12.7156 | | RL23 | | 7 | | - | | | 2 | | M9 | | | 2 | | (F39; F6) | |  |  |
|  |  | | | |  | | |  | | RL39 | |  | | - | | | / | | / | | | 2 | | F32 | |  |  |
|  |  | | | |  | | |  | | RL40 | |  | | - | | | 2 | | (M1; M16) | | | / | | / | |  |  |
|  |  | | | |  | | |  | | RL41 | |  | | - | | | 2 | | (M9; M22) | | | 2 | | (F37; F38) | |  |  |
|  |  | | | |  | | |  | | RL42 | |  | | - | | | 2 | | M9 | | | 2 | | (F6; F9) | |  |  |
|  |  | | | |  | | |  | | RL43 | |  | | - | | | 2 | | (M9; M22) | | | / | | / | |  |  |
|  |  | | | |  | | |  | | RL44 | |  | | - | | | 2 | | (M2; M9) | | | / | | / | |  |  |
| 10L | Castelporziano | | | | 41.7060 | | | 12.4193 | | RL32 | | 2 | | - | | | / | | / | | | 2 | | F6 | |  |  |
|  |  | | | |  | | |  | | RL34 | |  | | - | | | / | | / | | | 2 | | F39 | |  |  |
| 11L | Anagni | | | | 41.7379 | | | 13.1605 | | RL71 | | 2 | | - | | | 2 | | M9 | | | / | | / | |  |  |
|  |  | | | |  | | |  | | RL72 | |  | | - | | | 2 | | (M9; M21) | | | / | | / | |  |  |
| 12L | Bellegra | | | | 41.8775 | | | 13.0303 | | RL11 | | 5 | | - | | | 2 | | M9 | | | / | | / | |  |  |
|  |  | | | |  | | |  | | RL12 | |  | | - | | | / | | / | | | / | | / | |  |  |
|  |  | | | |  | | |  | | RL13 | |  | | - | | | 2 | | (M7; M9) | | | / | | / | |  |  |
|  |  | | | |  | | |  | | RL14 | |  | | - | | | 2 | | (M6; M9) | | | / | | / | |  |  |
|  |  | | | |  | | |  | | RL74 | |  | | - | | | 2 | | (M7; M15) | | | / | | / | |  |  |
| 13L | Olevano Romano | | | | 41.8611 | | | 13.0381 | | RL75 | | 1 | | - | | | 2 | | M9 | | | / | | / | |  |  |
| 15L | P. della Mola | | | | 42.1555 | | | 12.1492 | | RL30 | | 1 | | - | | | 2 | | M9 | | | 2 | | F39 | |  |  |
| 16L | Maccarese | | | | 41.8907 | | | 12.2760 | | RL15 | | 1 | | - | | | 2 | | (M9; M21) | | | 2 | | (F6; F7) | |  |  |
| 18L | M. della Tolfa | | | | 42.1369 | | | 11.9706 | | RL05 | | 1 | | - | | | / | | / | | | / | | / | |  |  |
| 19L | Foce Verde | | | | 41.3874 | | | 12.9240 | | RL18 | | 1 | | - | | | 2 | | (M9; M15) | | | 2 | | (F32; F34) | |  |  |
| 20L | Ventotene | | | | 40.480 | | | 13.260 | | RL207 | | 2 | | - | | | 2 | | M24 | | | / | | / | |  |  |
|  |  | | | |  | | |  | | RL208 | |  | | - | | | 2 | | (M9; M24) | | | / | | / | |  |  |
| 1T | Giannella | | | | 42.4608 | | | 11.1833 | | RT05 | | 2 | | - | | | 2 | | (M9; M22) | | | 2 | | F6 | |  |  |
|  |  | | | |  | | |  | | RT06 | |  | | - | | | 2 | | (M19; M21) | | | / | | / | |  |  |
| 2T | Feniglia | | | | 42.4179 | | | 11.2387 | | RT07 | | 2 | | - | | | 2 | | (M9; M20) | | | 2 | | F6 | |  |  |
|  |  | | | |  | | |  | | RT08 | |  | | - | | | 2 | | (M23; M30) | | | 2 | | F40 | |  |  |
| 4T | Pian della Rasa | | | | 44.035 | | | 11.053 | | RT10 | | 2 | | - | | | / | | / | | | / | | / | |  |  |
|  |  | | | |  | | |  | | RT11 | |  | | - | | | / | | / | | | / | | / | |  |  |
| 3M | M. Conca | | | | 43.8724 | | | 12.4982 | | RM15 | | 2 | | - | | | / | | / | | | 2 | | F8 | |  |  |
|  |  | | | |  | | |  | | RM17 | |  | | - | | | / | | / | | | / | | / | |  |  |
| 1E | Mesola | | | | 44.8555 | | | 12.2439 | | RE03 | | 5 | | - | | | 2 | | (M21; M28) | | | / | | / | |  |  |
|  |  | | | |  | | |  | | RE04 | |  | | - | | | 2 | | (M23; M31) | | | / | | / | |  |  |
|  |  | | | |  | | |  | | RE05 | |  | | - | | | 2 | | (M23; M26) | | | / | | / | |  |  |
|  |  | | | |  | | |  | | RE06 | |  | | - | | | 2 | | M27 | | | / | | / | |  |  |
|  |  | | | |  | | |  | | RE08 | |  | | - | | | / | | / | | | / | | / | |  |  |
| 2SA | Budoni | | | | 40.4200 | | | 9.4200 | | RSA10 | | 2 | | - | | | 2 | | M9 | | | / | | / | |  |  |
|  | | |  | | | |  | |  | | RSA11 | |  | | - 2 | | |  | | M9 | / | | | / |  |  |  |
|  | |  | |  | |  | | |  |  |  |  |  |  |  |  |  |  |  |  |  |  |  |  |  |  |  |
|  | |  |  |  |  |  |  |  |  |  |  |  |  |  |  |  |  |  |  |  |  |  |  |  |  |  |  |

**Table S2** Primers used in the study with relative references and cycling conditions. PCRs were conducted in a standard volume of 25 µL, containing 1 mM Tris-HCL (pH 9.0), 5 mM KCL, 0.15 mM MgCl_2_, 0.2 mM of each dNTP, 0.1 mM of both forward and reverse primer, and 0.5 units of *Taq* polymerase.

| **Gene** | **Primer** | **Primer sequence 5'- 3'** | **Reference** | | **PCR conditions** | |
| --- | --- | --- | --- | --- | --- | --- |
| *cytb* | CytF | TTTGGATCACTATTRGGCCTCTGCC | Senczuk et al., 2017 | Pre-denaturation: 94°C 2min Denaturation: 95°C 10sec Annealing: 55°C 20sec Elongation: 72°C 90sec Extension: 72°C 7min Cycles: 35 | |  |
|  | H15425 | GGTTTACAAGACCAGTGCTTT | Podnar et al., 2005 |  |  |  |
| *nd4* | ND4 | TACTTAAACTAGGGGGCTACGGCCTAATCCGCATT | Arèvalo et al., 1994 | Pre-denaturation: 94°C 3min Denaturation: 94°C 30sec Annealing: 54°C 30sec Elongation: 72°C 40sec Extension: 72°C 4min Cycles: 35 | |  |
|  | Leu | CATTACTTTTACTTGGATTTGCACCA |  |  |  |  |
| *mc1r* | MC1R-PF | GGCNGCCATYGTCAANAACCGGAACC | Buades et al., 2013 | Pre-denaturation: 92°C 5min Denaturation: 92°C 30sec Annealing: 56°C 30sec Elongation: 72°C 90sec Extension: 72°C 5min Cycles: 35 | |  |
|  | MC1R-PR | CTCCGRAAGGCRTAAATNATGGGGTCCAC |  |  |  |  |
| *β-fibint7* | FIB-B17U | CAGYACTTTYGAYAGAGACAAYGATGG | Prychitko et al., 1997 | Pre-denaturation: 94°C 3min Denaturation: 93°C 60sec Annealing: 56°C 30sec Elongation: 72°C 2min Extension: 72°C 10min Cycles: 33 | |  |
|  | FIB-B17L | TCCCCAGTAGTATCTGCCATTAGGGTT |  |  |  |  |

| **Locus** | **Multiplex** | **Primer sequence** | **Ta (°C)** | **Allele range** | **N° alleles** | **References** |
| --- | --- | --- | --- | --- | --- | --- |
| Pb73* | Mix1 | **FAM**-GCCCATGTCACTTCAGGTAGAAGC  GAAAACTAGGAGTTAGGGAGAAGG | 57 | 126-134 | 5(2) | Pinho et al., 2004 |
| Pli10* | Mix1 | **FAM**-TGACTTGTAGGGCTGGCTTT  AGCTGTTTCTCAGCTGTGGTC | 57 | 234-290 | 12(2) | Bloor et al., 2011 |
| Pli3 | Mix1 | **NED**-CATGAAGGGAGGCGATGTAT  GATCCCATTCTGTCTTGGAA | 58 | 226-258 | 9 | Bloor et al., 2011 |
| C9 | Mix1 | **VIC**-CATTGCTGGTTCTGGAGAAAG  CCTGATGAAGGGAAGTGGTG | 58 | 126-159 | 10(3) | Nembini and Opplinger, 2003 |
| Pli24** | Mix2 | **FAM**-CCACAAGGACTCAGGCTCTC  TCCCCCACTTAAGCATGTTC | 56 | 101-113 | 4 | Bloor et al., 2011 |
| Pli4** | Mix2 | **FAM**-TCAGTTCATGCATAAGGTCCA  TTCGGCATTTTTCTTCAGGT | 56 | 319-439 | 24(9) | Bloor et al., 2011 |
| Pli18 | Mix2 | **NED**-CAAGAATTGAGTTTGCAGTTCC  TGTCTGACAGAATGTGCTTCTC | 56 | 116-168 | 14(2) | Bloor et al., 2011 |
| Pb10 | Mix2 | **VIC**-AGTGGAATCGGCTGCAATAC  ACCAGTCCCAGGAATTTAGG | 58 | 202-226 | 6 | Pinho et al., 2004 |
| Pli21 | Mix3 | **FAM**-CCATTATGACCTTGCTGGTG  GAACTCTGGTGGCCCACAT | 58 | 106 | 1 | Bloor et al., 2011 |
| Lv-4-α | Mix3 | **PET**-CTGCAGGGAACAGAATTAACC | 60 | 95-121 | 10(2) | Boudjemadi et al., 1999 |
|  |  | CTGCCCAGAAAGCATTTCC |  |  |  |  |
| Lv-4-19 | Mix3 | **NED-**CTGTTGCTATTTTGTATGCTTAC  CTGCCCAGAAAGCATTTCC | 57 | 114-140 | 10(5) | Boudjemadi et al., 1999 |

**Table S3** PCRs were conducted in a standard volume of 25 μL, containing 1 mM Tris-HCL (pH 9.0), 5 mM KCL, 0.15 mM MgCl_2_, 0.2 mM of each dNTP, 0.1 mM of both forward and reverse primer, and 0.5 units of *Taq* polymerase. Number of asterisks indicate pair of loci amplifications.

**Table S4** Accession numbers of each gene fragment analysed in this study (bold) with relative haplotypes.

| **voucher** | **Accession number** | ***cytb* haplotype** | **Accession number** | ***nd4* haplotype** | **Accession number** | ***mc1r***  **haplotype(s)** | **Accession number** | ***β, fibint7* haplotype(s)** |
| --- | --- | --- | --- | --- | --- | --- | --- | --- |
| 6S_ 15 | KY064860 | - | **MH718506** | - | **/** | / | KY094514 | (F20; F25) |
| 6S_ 16 | KY064861 | - | **MH718507** | - | **/** | / | KY094515 | F20 |
| 14S_48 | KY064879 | - | **MH718508** | - | KY064618 | M9 | KY094517 | F20 |
| 14S_49 | KY064880 | - | **MH718509** | - | KY064619 | M9 | KY094518 | F20 |
| 15S_54 | KY064882 | - | **MH718510** | - | / | / | / | / |
| 16S_57 | KY064883 | - | **MH718511** | - | KY064623 | M9 | KY094520 | F21 |
| 19S_103 | KY064896 | - | **MH718512** | - | / | / | KY094524 | F24 |
| 19S_104 | KY064897 | - | **MH718513** | - | KY064637 | M9 | / | / |
| 19S_105 | KY064898 | - | **MH718514** | - | KY064638 | M9 | / | / |
| 25S_157 | KY064910 | - | **MH718515** | - | KY064650 | M9 | / | / |
| 25S_157b | KY064911 | - | **MH718516** | - | KY064651 | M9 | / | / |
| 31S_223 | KY064924 | - | **MH718517** | - | / | / | / | / |
| 36S_32 | KY064933 | - | **MH718518** | - | KY064676 | M9 | KY094529 | F20 |
| 36S_36 | **MH718664** | - | **MH718519** | - | KY064679 | (M9; M14) | KY094530 | (F20; F23) |
| 39S_165 | KY064945 | - | **MH718520** | - | KY064688 | (M9; M14) | KY094534 | (F21; F22) |
| 44S_169 | KY064954 | - | **MH718521** | - | KY064697 | (M9; M14) | KY094537 | F24 |
| 44S_170 | KY064955 | - | **MH718522** | - | KY064698 | M9 | / | / |
| 45S_196 | KY064956 | - | **MH718523** | - | / | / | / | / |
| 48S_190 | KY064963 | - | **MH718524** | - | / | / | / | / |
| 52S_06 | KY064973 | - | **MH718525** | - | KY064717 | M9 | KY094538 | (F20; F24) |
| 52S_07 | KY064974 | - | **MH718506** | - | KY064718 | (M9; M5) | / | / |
| 53S_05 | KY064978 | - | **MH718526** | - | KY064722 | (M9; M26) | KY094542 | F20 |
| 55S_09 | KY064980 | - | **MH718527** | - | KY064724 | M9 | / | / |
| 55S_11 | KY064981 | - | **MH718528** | - | / | / | KY094543 | (F20; F24) |
| 1CL_140 | KY064984 | - | **MH718529** | - | / | / | KY094547 | (F26; F27) |
| 2CL_141 | KY064985 | - | **MH718530** | - | / | / | KY094546 | (F20; F29) |
| 2CL_142 | KY064986 | - | **MH718531** | - | / | / | KY094548 | F18 |
| 4CL_113 | KY064988 | - | **MH718532** | - | KY064735 | M10 | KY094549 | (F13; F28) |
| 4CL_114 | KY064989 | - | **MH718533** | - | KY064736 | (M10; M13) | KY094550 | F16 |
| 5CL_136 | KY064990 | - | **MH718534** | - | / | / | KY094551 | (F30; F31) |
| 5CL_137 | KY064991 | - | **MH718535** | - | / | / | KY094552 | (F28; F15) |
| 8CL_148 | KY064996 | - | **MH718536** | - | / | / | KY094557 | F28 |
| 8CL_150 | KY064997 | - | **MH718537** | - | / | / | KY094558 | F28 |
| 11CL_119 | KY064998 | - | **MH718538** | - | / | / | / | / |
| 13CL_115 | KY065002 | - | **MH718539** | - | / | / | / | / |
| 13CL_116 | KY065003 | - | **MH718540** | - | / | / | / | / |
| 14CL_111 | KY065005 | - | **MH718541** | - | KY064747 | (M4; M9) | KY094563 | F14 |
| 14CL_112 | KY065006 | - | **MH718542** | - | KY064749 | (M10; M11) | KY094564 | F28 |
| 1P_ 06 | KY065018 | - | **MH718543** | - | / | / | KY094568 | F17 |
| 2P_ 07 | KY065019 | - | **MH718544** | - | KY064767 | M21 | / | / |
| 3P_ 08 | KY065020 | - | **MH718545** | - | KY064768 | M21 | KY094569 | F19 |
| 1C_ 14 | KY065027 | - | **MH718526** | - | KY064773 | M24 | KY094573 | F12 |
| 1C_ 15 | KY065028 | - | **MH718546** | - | KY064774 | M24 | KY094574 | F11 |
| 4C_ 10 | KY065034 | - | **MH718547** | - | **/** | / | **/** | / |
| 4C_ 11 | KY065035 | - | **MH718548** | - | KY064780 | M24 | KY094577 | F10 |
| 5C_ 05 | KY065037 | - | **MH718549** | - | KY064782 | (M21; M24) | / | / |
| 6C_ 02 | KY065039 | - | **MH718550** | - | KY064784 | (M21; M24) | / | / |
| 6C_ 03 | KY065040 | - | **MH718551** | - | / | / | KY094578 | (F8;F10) |
| 7C_ 19 | KY065041 | - | **MH718552** | - | / | / | KY094579 | F10 |
| 7C_ 20 | KY065042 | - | **MH718553** | - | / | / | KY094580 | (F10; F42) |
| 1L_ 120 | KY065057 | - | **MH718554** | - | KY064793 | (M9; M24) | / | / |
| 2L_ 46 | KY065058 | - | **MH718555** | - | KY064794 | (M9; M25) | / | / |
| 2L_ 47 | KY065059 | - | **MH718556** | - | KY064795 | (M9; M24) | KY094586 | F33 |
| 2L_ 48 | KY065060 | - | **MH718557** | - | KY064796 | (M9; M6) | / | / |
| 3L_ 19 | KY065061 | - | **MH718558** | - | KY064797 | M9 | / | / |
| 4L_ 20 | KY065067 | - | **MH718559** | - | KY064798 | (M8; M9) | / | / |
| 4L_ 50 | KY065063 | - | **MH718560** | - | KY064800 | M9 | KY094587 | F6 |
| 4L_ 51 | KY065064 | - | **MH718561** | - | KY064801 | M9 | KY094588 | (F35; F36) |
| 4L_ 52 | KY065065 | - | **MH718562** | - | KY064802 | (M8; M9) | **/** | / |
| 4L_ 53 | KY065066 | - | **MH718563** | - | KY064803 | (M8; M9) | **/** | / |
| 5L_161 | **MH718665** | - | **MH718564** | - | **MH704970** | (M30; M31) | / | / |
| 5L _163 | **MH718666** | - | **MH718565** | - | **MH704971** | (M9; M31) | **MH705031** | F3 |
| 5L _164 | **MH718667** | - | **MH718566** | - | **MH704972** | (M9; M31) | **MH705032** | F2 |
| 5L _166 | **MH718668** | - | **MH718567** | - | **MH704973** | M9 | **MH705033** | F1 |
| 5L _168 | **MH718669** | - | **MH718568** | - | **MH704974** | (M31; M33) | **MH705034** | F2 |
| 5L _169 | **MH718670** | - | **MH718569** | - | **MH704975** | (M9; M31) | **MH705035** | F2 |
| 5L _170 | **MH718671** | - | **MH718570** | - | **/** | / |  | / |
| 5L _171 | **MH718672** | - | **MH718571** | - | **MH704976** | (M3; M31) | **MH705036** | F2 |
| 6L_95 | **MH718673** | - | **MH718572** | - | **MH704977** | M31 | **MH705037** | (F2; F1) |
| 6L_96 | **MH718674** | - | **MH718573** | - | **MH704978** | (M23; M31) |  | / |
| 6L_97 | **MH718675** | - | **MH718574** | - | **MH704979** | (M23; M29) | **MH705038** | (F2; F1) |
| 6L_98 | **MH718676** | - | **MH718575** | - | **MH704980** | (M23; M31) | **MH705039** | F2 |
| 6L_99 | **MH718677** | - | **MH718546** | - | **MH704981** | (M23; M31) | **MH705040** | F1 |
| 6L_100 | **MH718678** | - | **MH718576** | - | **MH704982** | M23 | / | / |
| 6L_101 | **MH718679** | - | **MH718577** | - | **MH704983** | (M30; M31) | / | / |
| 6L_102 | **MH718680** | - | **MH718578** | - | **MH704984** | (M9; M31) | / | / |
| 6L_103 | **MH718681** | - | **MH718579** | - | **MH704985** | (M9; M23) | / | / |
| 6L_104 | **MH718682** | - | **MH718580** | - | **MH704986** | (M23; M31) | / | / |
| 6L_105 | **MH718683** | - | **MH718581** | - | **MH704987** | M23 | / | / |
| 6L_106 | **MH718684** | - | **MH718582** | - | **MH704988** | (M15; M19) | **MH705041** | F2 |
| 6L_107 | **MH718685** | - | **MH718583** | - | **MH704989** | M23 | / | / |
| 6L_109 | **MH718686** | - | **MH718584** | - | **MH704990** | (M17; M23) | **MH705042** | F1 |
| 6L_110 | **MH718687** | - | **MH718585** | - | **MH704991** | (M29; M30) | **MH705043** | F2 |
| 6L_111 | **MH718688** | - | **MH718586** | - | **MH704992** | M31 | / | / |
| 6L_113b | **MH718689** | - | **MH718587** | - | **MH704993** | (M9; M31) | / | / |
| 6L_114 | **MH718690** | - | **MH718588** | - | **MH704994** | (M9; M16) | / | / |
| 6L_117 | **MH718691** | - | **MH718589** | - | **MH704995** | M31 | / | / |
| 6L_119 | **MH718692** | - | **MH718590** | - | **MH704996** | M23 | / | / |
| 7aL_08 | **MH718693** | - | **MH718591** | - | **MH704997** | (M31; M33) | **MH705044** | F2 |
| 7aL_09 | **MH718694** | - | **MH718592** | - | **MH704998** | M33 | **MH705045** | F2 |
| 7aL_10 | **MH718695** | - | **MH718593** | - | **MH704999** | (M31; M33) | / | / |
| 7aL_28 | **MH718696** | - | **MH718594** | - | **MH705000** | M31 | **MH705046** | F1 |
| 7aL_29 | **MH718697** | - | **MH718595** | - | **MH705001** | (M31; M32) | / | / |
| 7bL_55 | **MH718698** | - | **MH718596** | - | **MH705002** | (M9; M33) | / | / |
| 7bL_57 | **MH718699** | - | **MH718597** | - | **MH705003** | (M33; M34) | **MH705047** | (F1; F3) |
| 7bL_58 | **MH718700** | - | **MH718598** | - | **MH705004** | (M9; M33) | **MH705048** | F41 |
| 7bL_59 | **MH718701** | - | **MH718599** | - | **MH705005** | M33 | **MH705049** | F2 |
| 7bL_60 | **MH718702** | - | **MH718600** | - | **MH705006** | (M9; M33) | **MH705050** | F2 |
| 7bL_61 | **MH718703** | - | **MH718601** | - | **MH705007** | M33 | **MH705051** | F2 |
| 7bL_62 | **MH718704** | - | **MH718602** | - | **MH705008** | M31 | / | / |
| 7bL_63 | **MH718705** | - | **MH718603** | - | **MH705009** | (M9; M31) | **MH705052** | F41 |
| 7bL_64 | **MH718706** | - | **MH718604** | - | **MH705010** | (M9; M33) | / | / |
| 7bL_65 | **MH718707** | - | **MH718605** | - | **MH705011** | (M31; M33) | **MH705053** | F3 |
| 7bL_67 | **MH718708** | - | **MH718606** | - | **MH705012** | (M23;M31) | **MH705054** | F3 |
| 7bL_68 | **MH718709** | - | **MH718607** | - | **MH705013** | M31 | / | / |
| 7bL_69 | **MH718710** | - | **MH718608** | - | **MH705014** | M31 | / | / |
| 7cL_130 | **MH718711** | - | **MH718609** | - | **MH705015** | (M31; M33) | / | / |
| 7cL_132 | **MH718712** | - | **MH718610** | - | **MH705016** | (M31; M33) | **MH705056** | (F4; F41) |
| 7cL_133 | **MH718713** | - | **MH718611** | - | **MH705017** | (M31; M21) | **MH705055** | F41 |
| 7cL_134 | **MH718714** | - | **MH718612** | - | **MH705018** | (M9; M33) | **MH705057** | F3 |
| 7cL_135 | **MH718715** | - | **MH718613** | - | **/** | / | / | / |
| 8L_136 | **MH718716** | - | **MH718614** | - | **MH705019** | M30 | / | / |
| 8L_138 | **MH718717** | - | **MH718615** | - | **MH705020** | M30 | / | / |
| 8L_139 | **MH718718** | - | **MH718616** | - | **MH705021** | M9 | / | / |
| 8L_140 | **MH718719** | - | **MH718617** | - | **MH705022** | (M30; M33) | **MH705058** | F3 |
| 8L_141 | **MH718720** | - | **MH718618** | - | **MH705023** | M31 | **MH705059** | F2 |
| 8L_142 | **MH718721** | - | **MH718619** | - | **MH705024** | (M30; M31) | **MH705060** | F2 |
| 8L_143 | **MH718722** | - | **MH718620** | - | **MH705025** | (M9; M31) | **MH705061** | F2 |
| 8L_144 | **MH718723** | - | **MH718621** | - | **MH705026** | M9 | **MH705062** | (F2; F1) |
| 8L_145 | **MH718724** | - | **MH718622** | - | **MH705027** | (M17; M18) | **MH705063** | (F2; F3) |
| 8L_146 | **MH718725** | - | **MH718623** | - | **MH705028** | M9 | / | / |
| 8L_148 | **MH718665** | - | **MH718624** | - | **MH705029** | (M31; M32) | **MH705064** | (F5; F3) |
| 8L_149 | **MH718666** | - | **MH718625** | - | **MH705030** | (M9; M31) | / | / |
| 9L_ 23 | KY065068 | - | **MH718626** | - | KY064804 | M9 | KY094589 | (F39; F6) |
| 9L_ 39 | KY065069 | - | **MH718627** | - | / | / | KY094590 | F32 |
| 9L_ 40 | KY065070 | - | **MH718628** | - | KY064806 | (M1; M16) | / | / |
| 9L_ 41 | KY065071 | - | **MH718629** | - | KY064807 | (M9; M22) | KY094591 | (F37; F38) |
| 9L_ 42 | **MH718726** | - | **MH718630** | - | KY064808 | M9 | KY094592 | (F6; F9) |
| 9L_ 43 | KY065072 | - | **MH718631** | - | KY064809 | (M9; M22) | / | / |
| 9L_ 44 | KY065073 | - | **MH718632** | - | KY064810 | (M2; M9) | / | / |
| 10L_32 | KY065074 | - | **MH718633** | - | / | / | KY094593 | F6 |
| 10L_34 | KY065075 | - | **MH718634** | - | / | / | KY094594 | F39 |
| 11L_71 | KY065076 | - | **MH718635** | - | KY064813 | M9 | / | / |
| 11L_72 | KY065077 | - | **MH718606** | - | KY064814 | (M9; M21) | / | / |
| 12L_11 | KY065078 | - | **MH718607** | - | KY064815 | M9 | / | / |
| 12L_12 | KY065079 | - | **MH718636** | - | / | / | / | / |
| 12L_13 | KY065080 | - | **MH718637** | - | KY064816 | (M7; M9) | / | / |
| 12L_14 | KY065081 | - | **MH718638** | - | KY064817 | (M6; M9) | / | / |
| 12L_74 | KY065082 | - | **MH718639** | - | KY064818 | (M7; M15) | / | / |
| 13L_75 | KY065083 | - | **MH718640** | - | KY064819 | M9 | / | / |
| 15L_30 | KY065084 | - | **MH718641** | - | KY064820 | M9 | KY094595 | F39 |
| 16L_15 | KY065085 | - | **MH718642** | - | KY064821 | (M9; M21) | KY094596 | (F6; F7) |
| 18L_05 | KY065086 | - | **MH718643** | - | / | / | / | / |
| 19L_18 | KY065087 | - | **MH718644** | - | KY064822 | (M9; M15) | KY094597 | (F32; F34) |
| 20L_209 | **MH718727** | - | **MH718645** | - | **MH718662** | M24 | / | / |
| 20L_208 | **MH718728** | - | **MH718646** | - | **MH718663** | (M9; M24) | / | / |
| 1T_ 05 | KY065093 | - | **MH718647** | - | KY064828 | (M9; M22) | KY094599 | F6 |
| 1T_ 06 | KY065094 | - | **MH718648** | - | KY064829 | (M19; M21) | / | / |
| 2T_ 07 | KY065096 | - | **MH718649** | - | KY064831 | (M9; M20) | KY094600 | F6 |
| 2T_ 08 | KY065097 | - | **MH718650** | - | KY064832 | (M23; M30) | KY094601 | F40 |
| 4T_ 10 | KY065098 | - | **MH718651** | - | / | / | / | / |
| 4T_ 11 | KY065099 | - | **MH718652** | - | / | / | / | / |
| 3M_15 | KY065106 | - | **MH718653** | - | / | / | KY094603 | F8 |
| 3M_17 | KY065107 | - | **MH718654** | - | / | / | **/** | / |
| 1E_ 03 | KY065111 | - | **MH718655** | - | KY064837 | (M21; M28) | **/** | / |
| 1E_ 04 | KY065112 | - | **MH718656** | - | KY064838 | (M23; M31) | **/** | / |
| 1E_ 05 | KY065113 | - | **MH718657** | - | KY064839 | (M23; M26) | **/** | / |
| 1E_ 06 | KY065114 | - | **MH718658** | - | KY064840 | M27 | **/** | / |
| 1E_ 08 | KY065115 | - | **MH718659** | - | / | / | **/** | / |
| 2SA_10 | KY065116 | - | **MH718660** | - | KY064729 | M9 | **/** | / |
| 2SA_11 | KY065117 | - | **MH718661** | - | KY064730 | M9 | **/** | / |

**Table S5.** Uncorrected pairwise *p-*distance calculated for each mtDNA clade obtained by the phylogenetic reconstruction.

| **Clade** | **P** | **T** | **A1** | **A2** | **C1** | **C2** | **S** |
| --- | --- | --- | --- | --- | --- | --- | --- |
| **P** | - |  |  |  |  |  |  |
| **T** | 0.075 | - |  |  |  |  |  |
| **A1** | 0.070 | 0.057 | - |  |  |  |  |
| **A2** | 0.063 | 0.047 | 0.025 | - |  |  |  |
| **C1** | 0.097 | 0.87 | 0.080 | 0.079 | - |  |  |
| **C2** | 0.073 | 0.080 | 0.076 | 0.070 | 0.066 | - |  |
| **S** | 0.073 | 0.077 | 0.079 | 0.066 | 0.054 | 0.051 | - |

**Table S6** Genetic characteristics of each microsatellite loci. N = number of individuals; R = size range of alleles in base pairs (bp); N_A_ = number of alleles, brackets indicate the number of private alleles; H_E_ = expected heterozygosity; H_O_ = observed heterozygosity; F_IS_ = inbreeding coefficient; N_T_ = total number of alleles, brackets indicate the number of private alleles.

|  |  | **ZA** | **GA** | **PO** | **FM** | **PA** | **Total** |
| --- | --- | --- | --- | --- | --- | --- | --- |
| **Pb73** | N | 5 | 20 | 19 | 5 | 11 | 60 |
|  | R | 126-132 | 126-134 | 126-136 | 126-134 | 126-134 | 126-134 |
|  | N_A_ | 2 | 3 | 5(2) | 3 | 3 | 5(2) |
|  | H_E_ | 0.3200 | 0.5837 | 0.5609 | 0.5400 | 0.5331 | 0.6520 |
|  | H_O_ | 0.4000 | 0.6000 | 0.6316 | 0.6000 | 0.7273 | 0.592 |
|  | F_IS_ | -0.143 | -0.002 | -0.099 | -0.000 | -0.183 | -0.096 |
| **Pli10** | N | 5 | 20 | 18 | 5 | 11 | 59 |
|  | R | 250-266 | 238-282 | 238-290 | 234-278 | 234-274 | 234-290 |
|  | N_A_ | 4 | 10(1) | 10(1) | 5 | 8 | 12(2) |
|  | H_E_ | 0.5800 | 0.8413 | 0.8009 | 0.7800 | 0.8058 | 0.8625 |
|  | H_O_ | 0.6000 | 0.8500 | 0.8333 | 0.8000 | 0.8182 | 0.780 |
|  | F_IS_ | 0.077 | 0.015 | -0.012 | 0.086 | 0.032 | 0.021 |
| **Pli3** | N | 6 | 20 | 18 | 5 | 11 | 60 |
|  | R | 238-254 | 226-250 | 230-258 | 226-246 | 230-258 | 226-258 |
|  | N_A_ | 5 | 6 | 8 | 3 | 8 | 9 |
|  | H_E_ | 0.7361 | 0.6475 | 0.8241 | 0.5600 | 0.8388 | 0.8559 |
|  | H_O_ | 0.8333 | 0.7500 | 0.7778 | 0.4000 | 0.7273 | 0.698 |
|  | F_IS_ | -0.042 | -0.133 | 0.085 | 0.385 | 0.179 | 0.049 |
| **C9** | N | 6 | 20 | 19 | 5 | 11 | 61 |
|  | R | 135-156 | 135-150 | 135-159 | 126-156 | 135-156 | 126-159 |
|  | N_A_ | 5 | 5 | 9(2) | 3(1) | 5 | 10(3) |
|  | H_E_ | 0.7500 | 0.7462 | 0.8366 | 0.5400 | 0.7645 | 0.8568 |
|  | H_O_ | 0.8333 | 0.8000 | 0.7895 | 0.2000 | 0.6364 | 0.652 |
|  | F_IS_ | -0.020 | -0.046 | 0.083 | 0.692 | 0.213 | 0.095 |
| **Pli24** | N | 6 | 20 | 19 | 5 | 11 | 61 |
|  | R | 101-109 | 101-109 | 101-113 | 105-113 | 101-109 | 101-113 |
|  | N_A_ | 3 | 2 | 3 | 3 | 3 | 4 |
|  | H_E_ | 0.2917 | 0.0950 | 0.3144 | 0.5800 | 0.4298 | 0.4084 |
|  | H_O_ | 0.0333 | 0.1000 | 0.3684 | 0.8000 | 0.5455 | 0.429 |
|  | F_IS_ | -0.053 | -0.027 | -0.145 | -0.280 | -0.224 | -0.176 |
| **Pli4** | N | 6 | 20 | 19 | 5 | 10 | 60 |
|  | R | 331-387 | 323-411 | 323-439 | 319-387 | 323-407 | 319-439 |
|  | N_A_ | 3 | 15(5) | 18(3) | 5(1) | 11 | 24(9) |
|  | H_E_ | 0.4028 | 0.8825 | 0.9169 | 0.7400 | 0.8850 | 0.9457 |
|  | H_O_ | 0.1667 | 0.9500 | 0.9474 | 0.4000 | 1.0000 | 0.693 |
|  | F_IS_ | 0.643 | -0.051 | -0.006 | 0.543 | -0.078 | 0.044 |
| **Pli18** | N | 6 | 20 | 19 | 5 | 11 | 61 |
|  | R | 124-160 | 120-168 | 116-168 | 120-132 | 128-152 | 116-168 |
|  | N_A_ | 6 | 12(1) | 13(1) | 4 | 7 | 14(2) |
|  | H_E_ | 0.7917 | 0.8812 | 0.8892 | 0.6400 | 0.7893 | 0.9244 |
|  | H_O_ | 0.8333 | 0.9500 | 1.0000 | 0.6000 | 0.7273 | 0.822 |
|  | F_IS_ | 0.038 | -0.052 | -0.098 | 0.172 | 0.126 | -0.013 |
| **Pb10** | N | 5 | 20 | 19 | 5 | 10 | 59 |
|  | R | 206-222 | 202-226 | 206-226 | 206-222 | 202-222 | 202-226 |
|  | N_A_ | 3 | 6 | 5 | 3 | 4 | 6 |
|  | H_E_ | 0.5800 | 0.7175 | 0.7078 | 0.5800 | 0.5100 | 0.7197 |
|  | H_O_ | 0.4000 | 0.7000 | 0.4037 | 0.8000 | 0.5000 | 0.575 |
|  | F_IS_ | 0.407 | 0.050 | 0.355 | -0.280 | 0.072 | 0.165 |
| **Lv19** | N | 5 | 19 | 16 | 5 | 9 | 54 |
|  | R | 120-140 | 120-128 | 120-130 | 126-128 | 114-136 | 114-140 |
|  | N_A_ | 6(2) | 2 | 5 | 2 | 7(3) | 10(5) |
|  | H_E_ | 0.8000 | 0.2285 | 0.6660 | 0.4800 | 0.7963 | 0.8049 |
|  | H_O_ | 0.4000 | 0.1579 | 0.0625 | 0.000 | 0.1111 | 0.146 |
|  | F_IS_ | 0.579 | 0.333 | 0.912 | 1.000 | 0.875 | 0.775 |
| **Lv4a** | N | 6 | 20 | 19 | 5 | 11 | 61 |
|  | R | 95-115 | 111-117 | 101-117 | 105-109 | 101-121 | 95-121 |
|  | N_A_ | 4(1) | 4 | 8 | 2 | 6(1) | 10(2) |
|  | H_E_ | 0.6528 | 0.6912 | 0.7922 | 0.3200 | 0.7851 | 0.9028 |
|  | H_O_ | 0.1667 | 0.5000 | 0.5789 | 0.0000 | 0.1818 | 0.285 |
|  | F_IS_ | 0.783 | 0.300 | 0.294 | 1.000 | 0.787 | 0.476 |
| **Total** | N | 6 | 20 | 19 | 5 | 11 | / |
|  | N_T_ | 41(2) | 65(7) | 84(9) | 33(2) | 62(3) | / |
|  | H_E_ | 0.5905 | 0.6315 | 0.7309 | 0.5760 | 0.7138 | / |
|  | H_O_ | 0.4967 | 0.6358 | 0.6463 | 0.4600 | 0.5975 | / |
|  | F_IS_ | 0.254 | 0.019 | 0.143 | 0.306 | 0.212 | / |

**Table S7.** P values for deviation from Hardy-Weinberg equilibrium. Bonferroni correction *p*<0.001.

|  | **ZA** | **GA** | **PO** | **FM** | **PA** |
| --- | --- | --- | --- | --- | --- |
| **Pb73** | 1.0000 | 0.7684 | 0.9294 | 0.6188 | 0.3713 |
| **Pli10** | 0.6185 | 0.6060 | 0.6538 | 0.2883 | 0.5086 |
| **Pli3** | 1.0000 | 0.8518 | 0.4246 | 002379 | 0.2243 |
| **C9** | 0.0780 | 0.4735 | 0.7919 | 0.4979 | 0.185 |
| **Pli24** | 1.0000 | 1.0000 | 1.0000 | 0.6201 | 1.0000 |
| **Pli4** | 0.0911 | 0.9777 | 0.6681 | 0.0224 | 1.0000 |
| **Pli18** | 0.8777 | 0.9025 | 0.8208 | 0.3033 | 0.3976 |
| **Pb10** | 0.3654 | 0.7863 | 0.1833 | 0.6182 | 1.0000 |
| **Lv19** | 0.0096 | 0.2587 | **<0.0001** | 0.0477 | **<0.0001** |
| **Lv4a** | 0.0046 | 0.0714 | 0.0058 | 0.1114 | **<0.0001** |

**References**

Arèvalo, E., Davis, S. K., & Sites Jr, J. W. (1994). Mitochondrial DNA sequence divergence and phylogenetic relationships among eight chromosome races of the *Sceloporus grammicus* complex (Phrynosomatidae) in central Mexico. *Systematic Biology*, **43**(3), 387-418.

Buades, J. M., Rodríguez, V., Terrasa, B., Perez-Mellado, V., Brown, R. P., Castro, J. A., & Ramon, M. M. (2013). Variability of the mc1r Gene in Melanic and Non-Melanic *Podarcis lilfordi* and *Podarcis pityusensis* from the Balearic Archipelago. *PloS one*, **8**(1), e53088.

Bloor, P., Rodríguez, V., Terrasa, B., Brown, R. P., Pérez-Mellado, V., Castro, J. A., & Ramon, M. M. (2011). Polymorphic microsatellite loci for the Balearic Island Lizard *Podarcis lilfordi* (Squamata: Lacertidae). Conservation Genetics Resources, **3**(2), 323-325.

Boudjemadi, K., Martin, O., Simon, J. C., & Estoup, A. (1999). Development and cross-species comparison of microsatellite markers in two lizard species, *Lacerta vivipara* and Podarcis muralis. *Molecular Ecology*, **8**(3), 518-520.

Nembrini, M., & Oppliger, A. (2003). Characterization of microsatellite loci in the wall lizard *Podarcis muralis* (Sauria: Lacertidae). *Molecular Ecology Resources*, **3**(1), 123-124.

Pinho, C., Sequeira, F., Godinho, R., Harris, D. J., & Ferrand, N. (2004). Isolation and characterization of nine microsatellite loci in *Podarcis bocagei* (Squamata: Lacertidae). *Molecular Ecology Resources*, **4**(2), 286-288.

Podnar, M., Mayer, W., & Tvrtković, N. (2005). Phylogeography of the Italian wall lizard, *Podarcis sicula*, as revealed by mitochondrial DNA sequences. *Molecular Ecology*, **14**(2), 575-588.

Prychitko, T. M., & Moore, W. S. (1997). The utility of DNA sequences of an intron from the β-fibrinogen gene in phylogenetic analysis of woodpeckers (Aves: Picidae). *Molecular phylogenetics and evolution*, **8**(2), 193-204.

Senczuk, G., Colangelo, P., De Simone, E., Aloise, G., & Castiglia, R. (2017). A combination of long term fragmentation and glacial persistence drove the evolutionary history of the Italian wall lizard *Podarcis siculus*. *BMC evolutionary biology*, **17**(1), 6.

**Figure S1.** Phylogenetic reconstruction of *Podarcis siculus* with MrBayes, based on concatenated data from *cytb* and *nd4.* The posterior probabilities of the main clades are shown at each node.


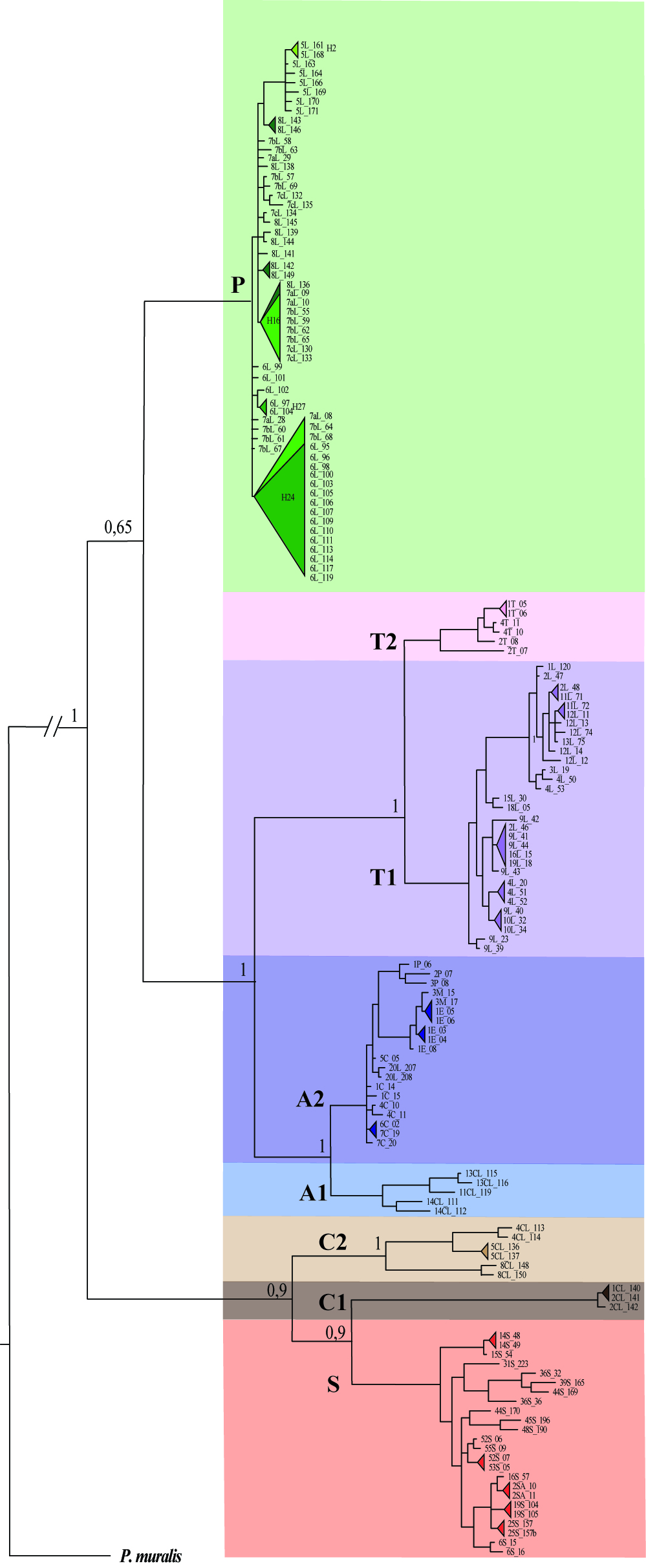


**Figure S2.** Values of BIC calculation versus number of clusters in the Discriminant Analysis of Principal Component (DAPC) for each analyzed dataset.


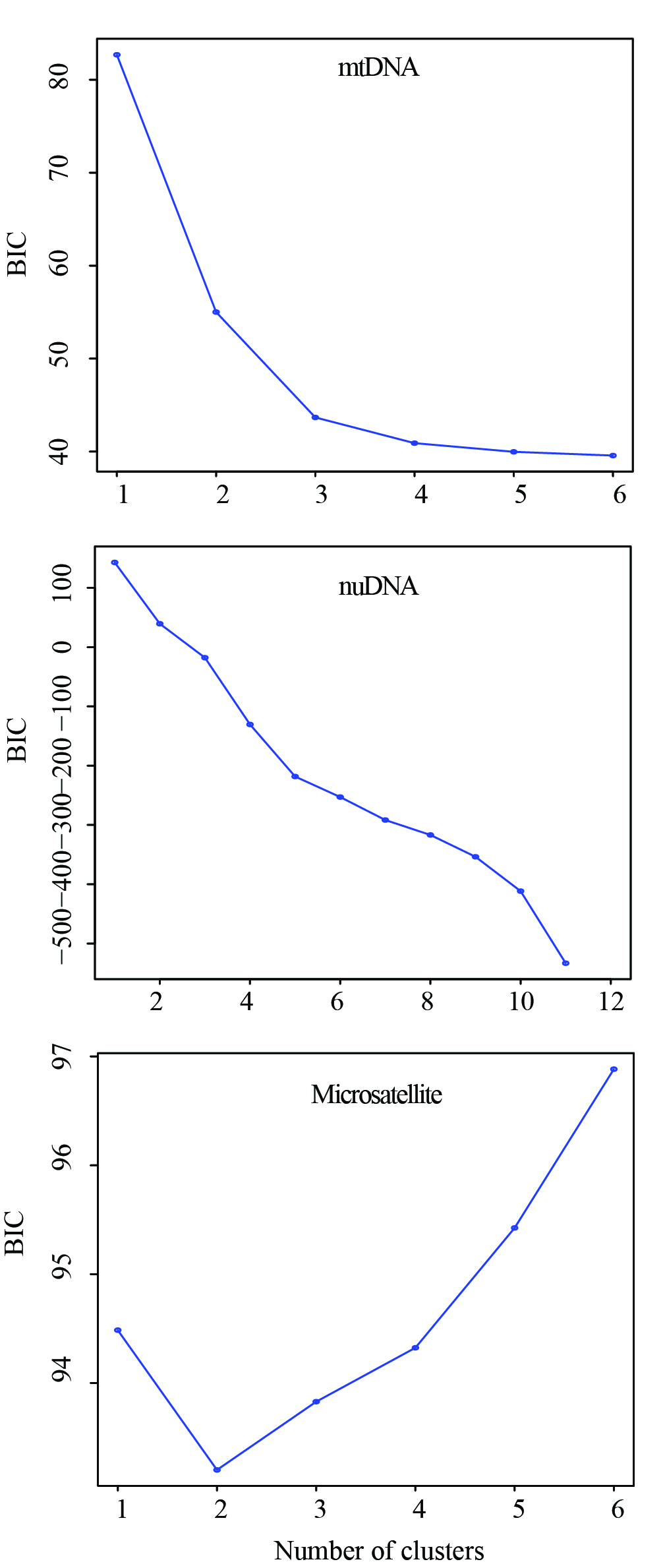
––

**Figure S3.** Evanno method in STRUCTURE HARVESTER (above) and the results of the Bayesian clustering analysis preformed in structure based on 10 microsatellite loci for 5 insular populations of *Podarcis siculus* (below). Group repartition at *K*=2 and *K*=4 is shown according to the rate of change of the likelihood function (deltaK). The genetic clusters are coded by distinct colors and each vertical line represents the membership probability of one individual to a genetic cluster. On the horizontal bar, each sampled island is shown: PO = Ponza; PA = Palmarola; GA = Gavi; ZA = Zannone; FM = Faraglioni della Madonna.

**
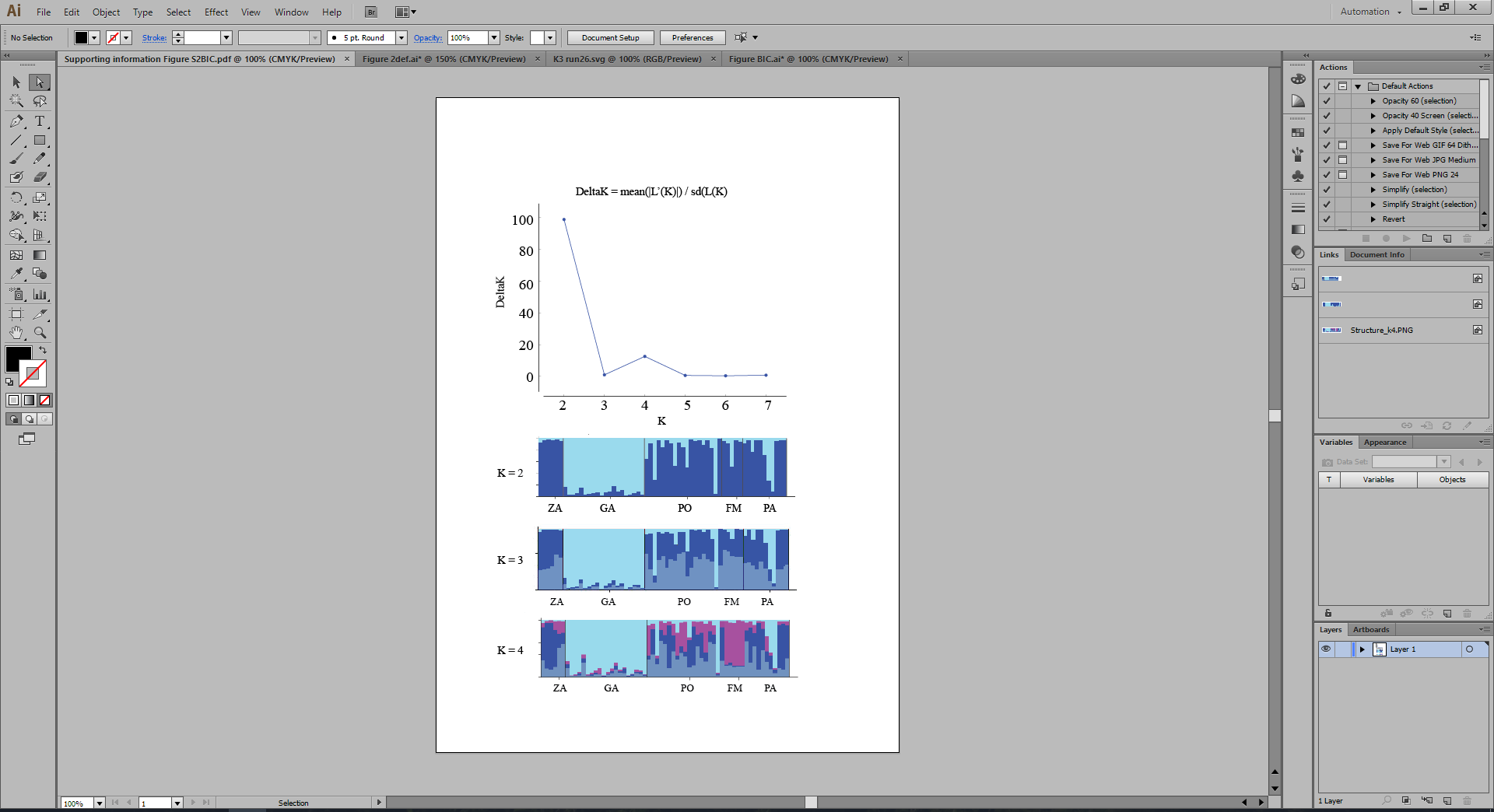
**
